# Supplementary figures and images for: Optical microscopy reveals the dynamic nature of B. pseudomallei morphology during β-lactam antimicrobial susceptibility testing
Source: BMC Microbiol. 2020 Jul 16;20:209. doi: 10.1186/s12866-020-01865-w (PMC7364477; doi:10.1186/s12866-020-01865-w)

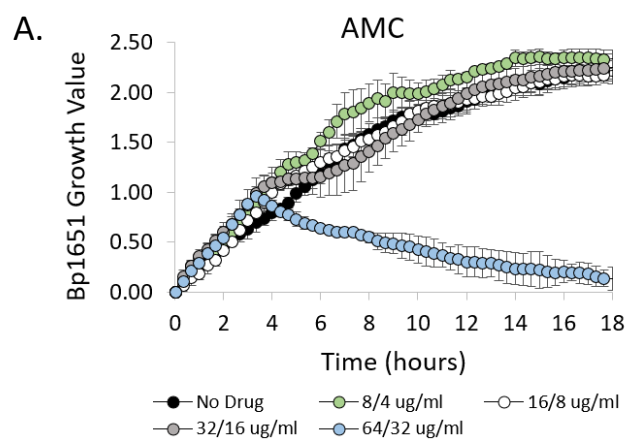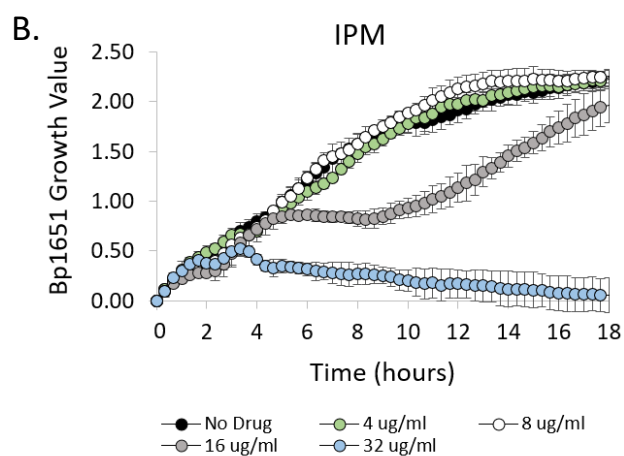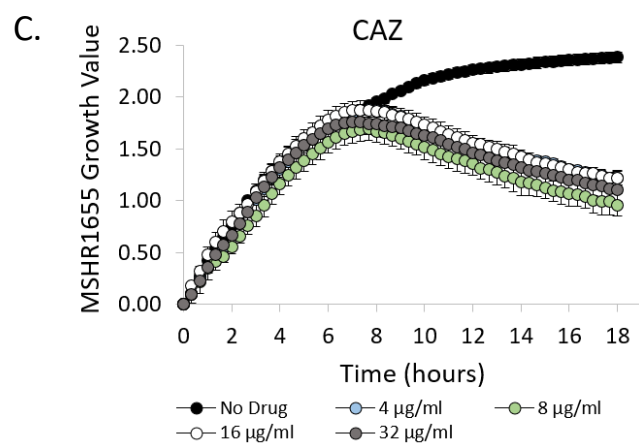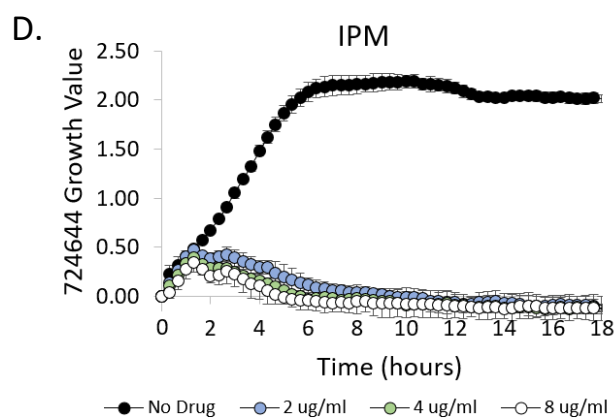

Supplement: Supplementary file 1 — Additional file 1: Figure S1. Growth kinetics ofB. pseudomalleistrains evaluated over 18 h in the presence and absence of β-lactams. Bp1651 exposed to AMC (A) and IPM (B), MSHR1655 exposed to CAZ (C) and 724644 exposed to IPM (D). Drug concentrations (μg/ml) corresponding to the CLSI breakpoint for susceptibility (green dots) and MICs (blue dots). Graphs represent the average ± standard deviation from triplicate samples. Amoxicillin-clavulanic acid (AMC), ceftazidime (CAZ), and imipenem (IPM) [file 12866_2020_1865_MOESM1_ESM.pdf]

6788 (AMC-S)

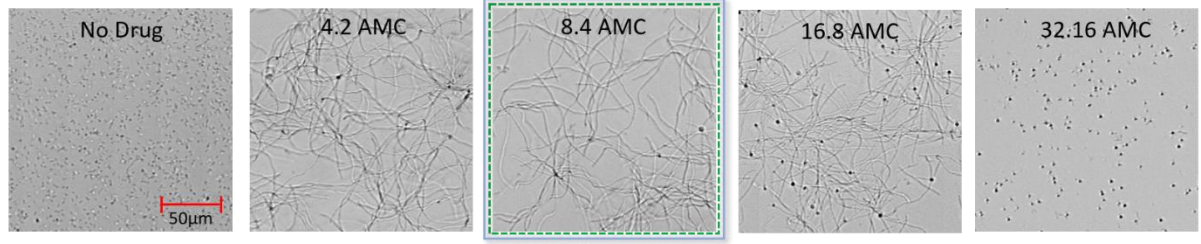

— MIC  
- - - CLSI breakpoint for susceptibility

Supplement: Supplementary file 2 — Additional file 2: Figure S2. Cell morphology ofB. pseudomallei6788 in the presence and absence of AMC (μg/ml). Optical screen images were captured after 6 h. Amoxicillin-clavulanic acid (AMC), CLSI breakpoints for susceptibility (green dotted line), MIC (blue line). [file 12866_2020_1865_MOESM2_ESM.pdf]
